# EZH1/2 alteration as a potential biomarker for immune checkpoint inhibitors across multiple cancer types

**DOI:** 10.1186/s12967-023-04759-3

**Published:** 2023-12-15

**Authors:** Huageng Huang, Xinyi Deng, Le Yu, He Huang, Zhao Wang, Huangming Hong, Tongyu Lin

**Affiliations:** 1 Department of Medical Oncology, State Key Laboratory of Oncology in South China, Guangdong Key Laboratory of Nasopharyngeal Carcinoma Diagnosis and Therapy, Guangdong Provincial Clinical Research Center for Cancer, Sun Yat-Sen University Cancer Center, 651 Dongfeng East Road, Guangzhou, 510060 People’s Republic of China; 2 Department of Dermatology, The First Affiliated Hospital of Guangzhou Medical University, Guangzhou, 510120 Guangdong People’s Republic of China; 3 Department of Medical Oncology, Senior Ward and Phase I Clinical Trial Ward, Sichuan Cancer Hospital & Institute, Sichuan Cancer Center, School of Medicine, University of Electronic Science and Technology of China, Chengdu, 610000 Sichuan People’s Republic of China

**Dear Editor,**

Immune checkpoint inhibitors (ICIs) have revolutionized the current cancer treatment paradigm, but quite a portion of patients fail to benefit from ICIs based on existing well-recognized predictors, including elevated tumor mutation burden (TMB), microsatellite instability-high (MSI-H) and programmed death-ligand 1 (PD-L1) overexpression [1, 2]. The enhancer of zeste homolog 2 (EZH2) and its highly related homolog EZH1 are essential epigenetic silencing factors [3]. Their genetic alteration can lead to carcinogenesis and suppressive tumor microenvironment through aberrant histone methyltransferase activity [4–6]. A correlation between EZH1/2 alteration and the therapeutic benefit of ICIs has been observed in several case reports [7, 8]. However, to our knowledge, a systematic analysis of EZH1/2 alteration frequency in tumors and its predictive value for ICI therapy has not yet been reported. In this work, we analyzed large multicohort data sets and demonstrated that EZH1/2 alteration is a promising predictive biomarker of ICI-positive efficacy across multiple cancer types.

The flow diagram of this study is depicted in Additional file 1: Fig. S1. We first probed the prevalence of EZH1/2 alteration in 65,853 patients with multiple cancer types from 213 non-redundant studies. EZH1/2 alteration was defined as mutation, structural variant, amplification, deep deletion and multiple alterations of EZH1 and/or EZH2. Then, the Memorial Sloan Kettering Cancer Center (MSKCC) cohort of 1661 ICI-treated patients with various cancer types sequenced by MSK-IMPACT assay was utilized to assess the relationship between EZH1/2 alteration and ICI outcomes. Another ICI treatment cohort consisting of 7 additional independently published studies with survival data served as a validation cohort to further validate the predictive function of EZH1/2 alteration on ICI efficacy. Patients’ characteristics at baseline were compared by T-test or Mann–Whitney U test (continuous variables) and χ^2^ test or Fisher’s exact test (categorical variables). The overall survival (OS) (calculated from the ICI treatment start date) was estimated by the Kaplan–Meier method and compared between groups (*P* values by log-rank test, HR [hazard ratio] and 95% confidence interval [CI] by Cox regression model). To elucidate whether the value of EZH1/2 alteration on ICI efficacy stems from the impact on prognosis, we compared the OS and progression-free survival (PFS) (calculated from the date of first diagnosis) of 10,968 non-ICI-treated patients from The Cancer Genome Atlas pan‑cancer cohort based on EZH1/2 alteration status. All analyses were performed using SPSS V.25.0 (IBM Corp) and were considered statistically significant if *P* < 0.05 (two-sided). Institutional board approval and patient informed consent were waived because all the clinical and alteration data were de-identified and publicly available online.

A total of 3.0% (1931/65,853) of patients with distinct cancer types harbored EZH1/2 alteration. As shown in Fig. 1, there were 24 cancers with an alteration frequency above 1%. Nonmelanoma skin cancer (8.9%), melanoma (8.5%), endometrial cancer (7.7%), ovarian cancer (6.2%) and leukemia/lymphoma (4.9%) have a relatively higher prevalence. EZH1/2 alterations were predominantly missense mutations, followed by truncation mutations (Additional file 2: Fig. S2).

**Fig. 1:**
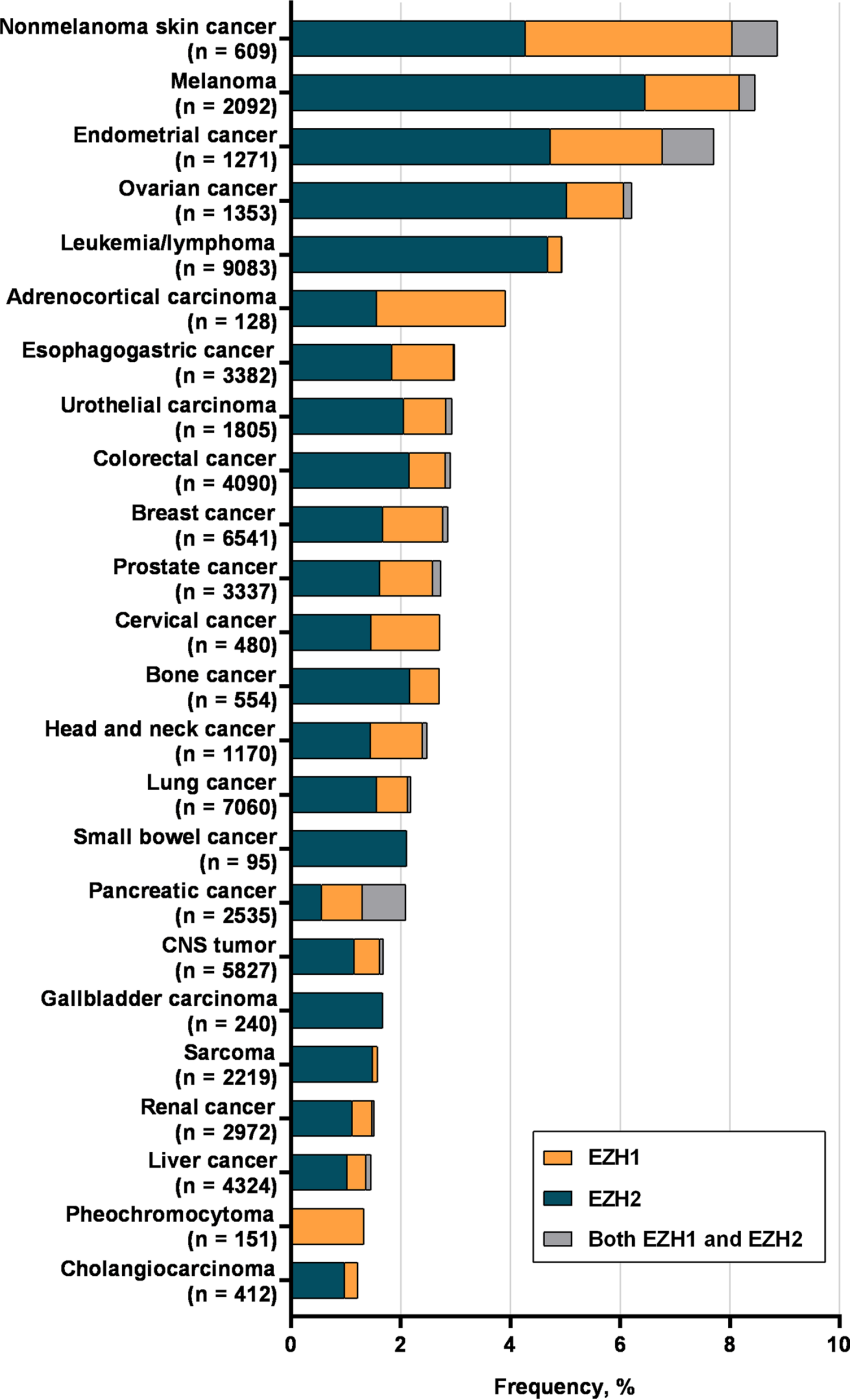
Prevalence of EZH1/2 alteration in 65,853 patients with different cancer types. CNS indicates the central nervous system

In the ICI treatment discovery and validation cohorts, baseline characteristics including age, sex, drug type and sample type were well balanced between the EZH1/2 altered and wild-type groups (all *P* > 0.05; Additional file 3: Table S1). Evaluation of the discovery cohort showed that median OS was significantly longer in EZH1/2 altered patients (n = 44) than in the wild-type population (n = 1617) (> 30.2 [not reach] vs. 18.0 months, HR = 0.596 [95% CI 0.357 to 0.994], *P* = 0.047; Fig. 2A). This relationship was stable in the multivariate-adjusted Cox model (HR = 0.551 [95% CI 0.330 to 0.920], adjusted *P* = 0.023; Additional file 4: Table S2). Notably, in the validation cohort, OS benefit remained more prominent in the EZH1/2 altered group (n = 51) than in the wild-type group (n = 886) (32.8 vs. 17.7 months, HR = 0.657 [95% CI 0.446 to 0.869], *P* = 0.034; Fig. 2B). After accounting for confounding factors, EZH1/2 alteration still independently predicted favorable OS outcomes (HR = 0.672 [95% CI 0.443 to 0.918], adjusted *P* = 0.041; Additional file 4: Table S2). These results suggest that EZH1/2 alteration is an independent predictor of positive ICI efficacy. In the non-ICI treatment cohort, there was no significant difference in OS or PFS between patients with EZH1/2 alteration (n = 454) and wild-type (n = 10,513), indicating that EZH1/2 alteration is not a prognostic factor (OS, HR = 0.923 [95% CI 0.781 to 1.090], *P* = 0.345; PFS, HR = 1.003 [95% CI 0.860 to 1.171], *P* = 0.965; Fig. 2C and D). Interestingly, patients harboring EZH1/2 alteration had a substantially higher median TMB than that of wild-type patients in the ICI treatment discovery and validation cohorts (both *P* < 0.001; Fig. 2E and F), as validated in the non-ICI treatment cohort (*P* < 0.001; Fig. 2G). In addition, EZH1/2 alteration was strongly correlated with the upregulation of genomic alterations associated with the DNA damage response pathway (*P* < 0.05; Fig. 2H).

**Fig. 2:**
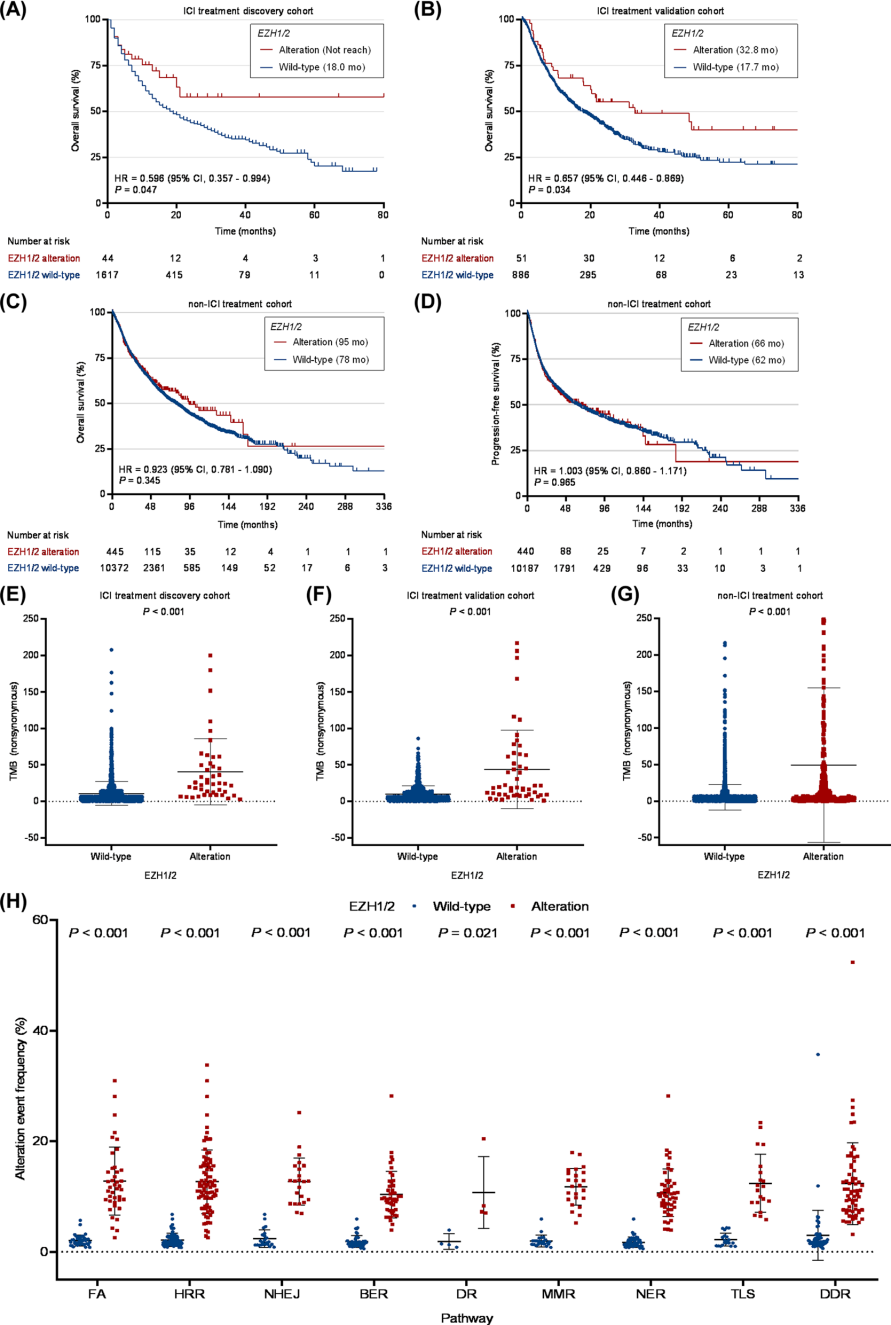
The role of EZH1/2 alteration in ICI therapy. Kaplan–Meier survival analysis comparing overall survival between EZH1/2 alteration and wild-type patients in the ICI treatment, **A** discovery cohort and **B** validation cohort. Kaplan–Meier survival analysis comparing, **C** overall survival and **D** progression-free survival between EZH1/2 alteration and wild-type patients in the non-ICI treatment cohort. Comparison of the TMB between the EZH1/2 alteration and wild-type groups in the ICI treatment, **E** discovery and **F** validation cohorts and **G** non-ICI treatment cohort. **H** Comparison of alteration event frequency associated with DDR pathway between EZH1/2 alteration and wild-type patients in the TCGA pan‑cancer cohort. ICI, immune checkpoint inhibitor; HR, hazard ratio; CI, confidence interval; TMB, tumor mutation burden; FA, Fanconi anemia; HRR, homologous recombination repair; NHEJ, nonhomologous end-joining; BER, base excision repair; DR, direct repair; MMR, mismatch repair; NER, nucleotide excision repair; TLS, translesion synthesis; DDR, DNA damage response; TCGA, The Cancer Genome Atlas

To the best of our knowledge, this is the first comprehensive study to report that EZH1/2 alteration was prevalent in a wide range of cancer types and could predict positive ICI outcomes. Our findings add great value in the application of screening for EZH1/2 alteration before ICI therapy, helping to avoid delays in effective treatment and the financial burden of no clinical benefit. More importantly, we reveal the possibility of individualized ICIs combined with EZH1/2 inhibitors for cancer treatment in the future.

Although the predictive value of EZH1/2 alteration was remarkable, one may be concerned about its relatively low average frequency. Actually, its scope of application falls in a pan-cancer setting like MSI-H, which occurs with a frequency of 2 ~ 4% of all cancers. However, MSI-H is particularly clustered in colorectal, endometrial and gastric cancers, while it is rarely detected in other cancers [9]. In this study, we showed that EZH1/2 alteration was also more common in endometrial cancer, as well as nonmelanoma skin cancer, melanoma, ovarian cancer, leukemia/lymphoma and others. Thus, there will still be a great number of EZH1/2 altered patients who will be screened and most likely to derive clinical benefit from ICIs. Furthermore, some existing biomarkers, such as PD-L1 and TMB, are either continuous variables with no universal cutpoints, or their expression varies widely across different assay platforms and methods [9]. In contrast, EZH1/2 alteration can be readily detected by next-generation sequencing, and its presence in the current analyses correlates closely with positive ICI response, elevated TMB and DNA repair deficiency. Therefore, we propose that EZH1/2 alteration should be considered alongside other known essential genes to expand the landscape of immuno-oncology genomic panels and integrate them into multiomics to fully enable precision immunotherapy.

In conclusion, this study provides clinical evidence that EZH1/2 alteration is a positive predictor of ICI outcomes across multiple cancer types. Due to data restrictions and limited sample size, further prospective studies and molecular mechanism exploration are warranted.

### Supplementary Information

**Additional file 1:** **Figure S1.** Flow diagram of the study. ICI, immune checkpoint inhibitor; MSKCC, Memorial Sloan Kettering Cancer Center; TCGA, The Cancer Genome Atlas; TMB, tumor mutation burden; DDR, DNA damage response.

**Additional file 2: Figure S2.** Protein domains and mutation location for EZH1/2 mutation. (A) EZH1, and (B) EZH2. The color of the circle indicates the corresponding mutation types. In the case of different mutation types at a single position, the color of the circle depends on the most frequent mutation type. Truncating mutation indicates nonsense, nonstop, frameshift deletion, frameshift insertion, and splice site.

**Additional file 3:** **Table S1.** Baseline characteristics of patients treated with immune checkpoint inhibitors in discovery and validation cohorts. WT, wild-type.

**Additional file 4:** **Table S2.** COX regression analyses of overall survival in patients treated with immune checkpoint inhibitors in discovery and validation cohorts. HR, hazard ratio; CI, confidence interval; WT, wild-type.

## Data Availability

All of the data we used in this study were publicly available from the cBioPortal database (https://www.cbioportal.org) and IMvigor210CoreBiologies website (http://research-pub.gene.com/IMvigor210CoreBiologies).
