# Supplementary figures and images for: EZH1/2 alteration as a potential biomarker for immune checkpoint inhibitors across multiple cancer types
Source: J Transl Med. 2023 Dec 15;21:913. doi: 10.1186/s12967-023-04759-3 (PMC10724995; doi:10.1186/s12967-023-04759-3)

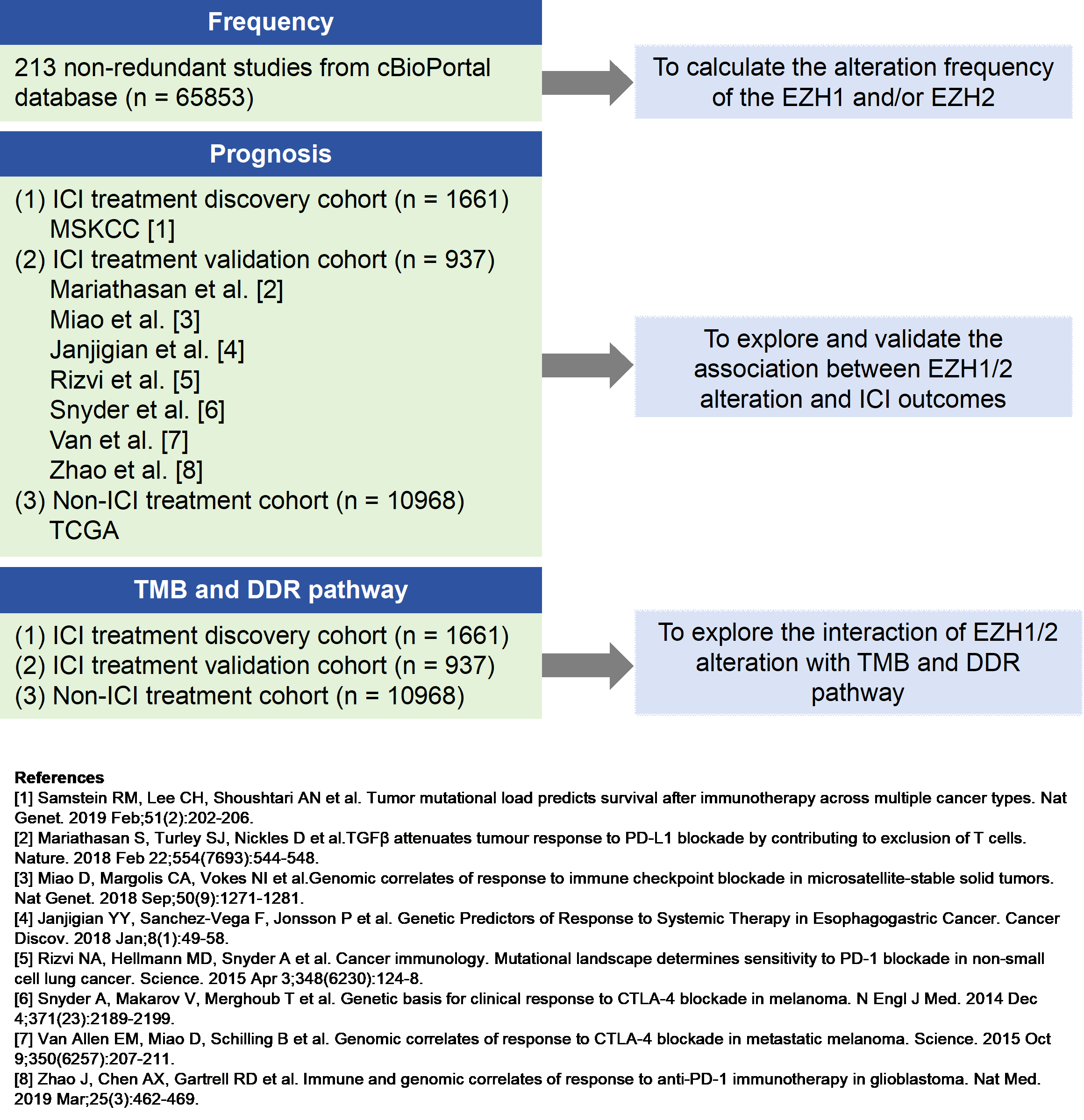

Supplement: Supplementary file 1 — Additional file 1: Figure S1. Flow diagram of the study. ICI, immune checkpoint inhibitor; MSKCC, Memorial Sloan Kettering Cancer Center; TCGA, The Cancer Genome Atlas; TMB, tumor mutation burden; DDR, DNA damage response. [file 12967_2023_4759_MOESM1_ESM.tif]

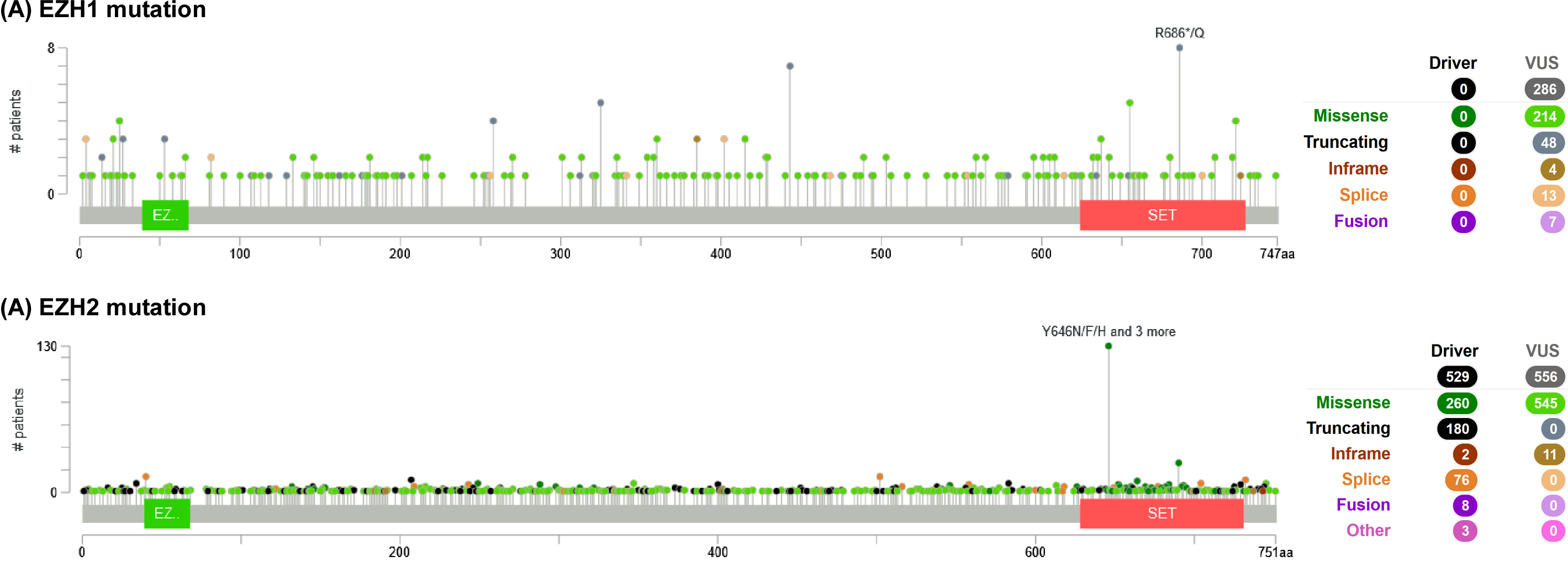

Supplement: Supplementary file 2 — Additional file 2: Figure S2. Protein domains and mutation location for EZH1/2 mutation. (A) EZH1, and (B) EZH2. The color of the circle indicates the corresponding mutation types. In the case of different mutation types at a single position, the color of the circle depends on the most frequent mutation type. Truncating mutation indicates nonsense, nonstop, frameshift deletion, frameshift insertion, and splice site. [file 12967_2023_4759_MOESM2_ESM.tif]
