# Supplementary material for: EZH1/2 alteration as a potential biomarker for immune checkpoint inhibitors across multiple cancer types
Source: J Transl Med. 2023 Dec 15;21:913. doi: 10.1186/s12967-023-04759-3 (PMC10724995; doi:10.1186/s12967-023-04759-3)
Supplement: Supplementary file 3 — Additional file 3: Table S1. Baseline characteristics of patients treated with immune checkpoint inhibitors in discovery and validation cohorts. WT, wild-type. [file 12967_2023_4759_MOESM3_ESM.docx]

**Table S1.** Baseline characteristics of patients treated with immune checkpoint inhibitors in discovery and validation cohorts. WT, wild-type.

| **Characteristics** | | **Discovery cohort** | | |  | **Validation cohort** | | |
| --- | --- | --- | --- | --- | --- | --- | --- | --- |
|  |  | **EZH1/2-WT group (n = 1617)** | **EZH1/2-altered group (n = 44)** | ***P* value** |  | **EZH1/2-WT group (n = 886)** | **EZH1/2-altered group (n = 51)** | ***P* value** |
| Age (%) | | | | | | | | |
|  | < 65 y | 900 (55.7) | 22 (50.0) | 0.456 |  | 358 (40.4) | 25 (49.0) | 0.053 |
|  | ≥ 65 | 717 (44.3) | 22 (50.0) |  |  | 203 (22.9) | 25 (49.0) |  |
|  | Missing | 0 (0) | 0 (0) |  |  | 325 (36.7) | 1 (2.0) |  |
| Sex (%) | | | | | | | | |
|  | Male | 1006 (62.2) | 28 (63.6) | 0.848 |  | 620 (70.0) | 33 (64.7) | 0.426 |
|  | Female | 611 (37.8) | 16 (36.4) |  |  | 266 (30.0) | 18 (35.3) |  |
| Drug type (%) | | | | | | | | |
|  | Mono | 1364 (84.4) | 42 (95.5) | 0.053 |  | 697 (78.7) | 49 (96.1) | 0.390 |
|  | Combo | 253 (15.6) | 2 (4.5) |  |  | 22 (2.5) | 0 (0) |  |
|  | Missing | 0 (0) | 0 (0) |  |  | 167 (18.8) | 2 (3.9) |  |
| Sample type (%) | | | | | | | | |
|  | Primary | 714 (44.2) | 17 (38.6) | 0.467 |  | 184 (20.8) | 2 (3.9) | 1.000 |
|  | Metastasis | 903 (55.8) | 27 (61.4) |  |  | 66 (7.4) | 0 (0) |  |
|  | Missing | 0 (0) | 0 (0) |  |  | 636 (71.8) | 49 (96.1) |  |
