# Supplementary material for: EZH1/2 alteration as a potential biomarker for immune checkpoint inhibitors across multiple cancer types
Source: J Transl Med. 2023 Dec 15;21:913. doi: 10.1186/s12967-023-04759-3 (PMC10724995; doi:10.1186/s12967-023-04759-3)
Supplement: Supplementary file 4 — Additional file 4: Table S2. COX regression analyses of overall survival in patients treated with immune checkpoint inhibitors in discovery and validation cohorts. HR, hazard ratio; CI, confidence interval; WT, wild-type. [file 12967_2023_4759_MOESM4_ESM.docx]

**Table S2**. COX regression analyses of overall survival in patients treated with immune checkpoint inhibitors in discovery and validation cohorts. HR, hazard ratio; CI, confidence interval; WT, wild-type.

| **Parameter** | | **Univariable analysis** | |  | **Multivariable analysis** | |
| --- | --- | --- | --- | --- | --- | --- |
|  |  | **HR (95% CI)** | ***P* value** |  | **HR (95% CI)** | ***P* value** |
| **Discovery cohort** | | | | | | |
|  | Age (< 65 y vs. ≥ 65 y) | 1.004 (0.876 - 1.152) | 0.951 |  | 0.978 (0.853 - 1.122) | 0.754 |
|  | Sex (male vs. female) | 1.134 (0.986 - 1.303) | 0.078 |  | 1.130 (0.983 - 1.299) | 0.085 |
|  | Drug type (mono vs. combo) | 0.561 (0.453 - 0.695) | < 0.001 |  | 0.554 (0.447 - 0.687) | < 0.001 |
|  | EZH1/2 (WT vs. altered) | 0.596 (0.357 - 0.994) | 0.047 |  | 0.551 (0.330 - 0.920) | 0.023 |
| **Validation cohort** | | | | | | |
|  | Age (< 65 y vs. ≥ 65 y) | 1.154 (0.930 - 1.433) | 0.194 |  | 1.125 (0.865 - 1.462) | 0.380 |
|  | Sex (male vs. female) | 0.955 (0.795 - 1.148) | 0.626 |  | 1.096 (0.832 - 1.443) | 0.516 |
|  | Drug type (mono vs. combo) | 0.570 (0.304 - 1.067) | 0.079 |  | 0.606 (0.284 - 1.294) | 0.196 |
|  | EZH1/2 (WT vs. altered) | 0.657 (0.446 - 0.869) | 0.034 |  | 0.672 (0.443 - 0.918) | 0.041 |
